# Evaluation of ultrasound technology to break seed dormancy of common lambsquarters (*Chenopodium album*)

**DOI:** 10.1002/fsn3.1547

**Published:** 2020-05-15

**Authors:** Ali Babaei‐Ghaghelestany, Mohammad Taghi Alebrahim, Dana R. MacGregor, Seyedeh Assieh Khatami, Rafat Hasani Nasab Farzaneh

**Affiliations:** ^1^ Department of Agronomy and Plant Breeding University of Mohaghegh Ardabili Ardabil Iran; ^2^ Department of Biointeractions and Crop Protection Rothamsted Research Harpenden UK

**Keywords:** common lambsquarters, germination percentage, seed dormancy, ultrasound technology

## Abstract

Although seed dormancy is advantageous for annual plants in the wild, unsynchronized germination in the laboratory leads to increased error in measurements. Therefore, techniques to promote and synchronize germination are routinely used. Ultrasound is one of the newest methods for breaking dormancy in weed seeds. We have investigated whether ultrasonic waves can be used to break seed dormancy of common lambsquarters (*Chenopodium album*), a highly competitive annual weed that leads to significant reduction of yields of corn, soybeans, and sugar beets. Ultrasonic waves with frequency of 35 kH were applied for 0 (control), 5, 10, 15, and 30 min using a completely randomized design. The results showed that the use of ultrasound waves generally enhanced the traits under investigation in the treated samples compared with the control sample. The maximum enhancement of germination percentage (180%), seedling dry weight (78%), and seedling vigor index I (271%) and II (392%) was seen in the common lambsquarters samples treated with ultrasound for 15 min and seedling length (40%) at 30 min compared with the control samples. Radical lengths were not statistically different from controls under any treatment and plumule length only increased marginally. These changes are reflected in seedling vigor index I and II measurements. For some of these traits, increasing the length of ultrasound treatment to 30 min had negative effects. These results demonstrate that ultrasound technology can be used as a quick, and efficient nondestructive method to break seed dormancy in common lambsquarters.

## Graphical abstract

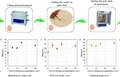

## 1 INTRODUCTION

To produce consistent high yields from agricultural systems, weed control is essential. Common lambsquarters (*Chenopodium album*) is one of the world's most problematic weeds (Moechnig, Stoltenberg, Boerboom, & Binning, 2003). Characteristics such as high growth rate, effective competition for resources and nutrients, high seed production, and seed germination under a wide range of environmental conditions means that common lambsquarters prevents crop growth and results in reduced crop yield (Schuster, Shoup, & Al‐Khatib, 2007). An important step to designing effective weed control is to understand the weed's biology and life cycle, especially the ecophysiological characteristics of its seeds (Long, Tan, Baskin, & Baskin, 2012).

The transition from seed to seedling is a key phenological transition and is under tight genetic and environmental control (Finch‐Savage & Leubner‐Metzger, 2006; Graeber, Nakabayashi, Miatton, Leubner‐Metzger, & Soppe, 2012). Seeds of annual plants like weeds frequently exhibit dormancy at maturity. This key adaptive trait is established during mid‐maturation of the seed within the mother plant (Alboresi et al., 2005; Karssen, Brinkhorst‐van der Swan, Breekland, & Koornneef, 1983), and its aim is to block seed germination other conditions that would otherwise be favorable for germination. Without seed dormancy, soil seed banks could not be established or maintained and is therefore essential for annual weeds like lambsquarters. Seed dormancy plays a role in weed survival, and the time of germination is an important phenomenon in the plant life cycle because the establishment of weeds depends on their seed germination ability. Seed dormancy in annual plants enables a large portion of the seed bank of weeds to correctly time germination with the seasons and/or to remain in the soil for a long time.

Although seed dormancy is advantageous for plants in the wild, it is a hindrance in the laboratory as varied germination rates and timings can alter seedling counts and sizes at a given time. Therefore, laboratory methods to uniformly break dormancy and promote germination are frequently used. Environmental conditions such as time since seed shed, light quality (sensed primarily by phytochromes), temperature, nutrient availability, humidity, and some gases break seed dormancy (Kępczyński & Sznigir, 2013; Penfield & King, 2009). In laboratory conditions, cold stratification, scarification, light and temperature alternations, or incubation with water and/or germination‐promoting solutions are typically used to break seed dormancy in weeds (Alebrahim, Rashedmihasel, Meighani, & Baghestani, 2011; Alshallash, 2018; Chu, Jusaitis, Aspinall, & Paleg, 1978; Fallahi, Babaei Ghaghelestany, Asadi Gakieh, & Hatami Gharah Ghovini, 2016; Humphries, Chauhan, & Florentine, 2018; Majd, Aghaie, Monfared, & Alebrahim, 2013; Tang et al., 2008). Of these, dry after‐ripening, and application of nitrate, cold, thiourea, water or nutrient solutions have shown to be effective in breaking dormancy in common lambsquarters (Chu et al., 1978; Cumming, 1963; Henson, 1970; Herron, 1953; Holm & Miller, 1972a, 1972b; Tang et al., 2008; Williams, 1962, 1963). However, these protocols are not all uniformly effective, and some require considerable time or resources. Therefore, inexpensive, nondestructive, and effective alternatives are required.

Ultrasound is a new physical method that involves the use of sound frequencies in the inaudible range (20–100 kHz) for interaction with materials. Ultrasound can change the state of the substance and even accelerate reactions. The presence of hard cell walls in some plants prevents the penetration of water and oxygen into the cell and reduces their germination potential. One of the reasons for the improvement in germination rate is the stimulation of the cellular wall of the seed by ultrasound. The advantages of the ultrasound technique are that it is simple, inexpensive, and environmentally friendly (Abbaspour‐Gilandeh, Kaveh, & Jahanbakhshi, 2019; Goussous, Samarah, Alqudah, & Othman, 2010; Kaveh, Jahanbakhshi, Abbaspour‐Gilandeh, Taghinezhad, & Moghimi, 2018). Ultrasound technology can also be used in the solid‐phase microextraction and the detection of bioactive compounds in plants (Asfaram, Sadeghi, Goudarzi, Kokhdan, & Salehpour, 2019). Researchers have discussed the use of ultrasound technology to stimulate germination of various seeds such as maize, barley, rice, and sunflower seeds and emphasized its importance (Ding et al., 2018; Rifna, Ramanan, & Mahendran, 2019; Sadeghianfar, Nazari, & Backes, 2019; Yaldagard, Mortazavi, & Tabatabaie, 2008). Liu et al. (2016) demonstrate that the use of ultrasound waves increased and improved the growth of tall fescue (*Festuca arundinacea*) and Russian wild rye (*Psathyrostaehys juncea* Nevski) seedlings. Likewise, germination of sesame (*Sesamum indicum* L.) seeds treated with ultrasound waves showed increased germination and growth (Shekari, Mustafavi, & Abbasi, 2015). Therefore, we set out to determine whether the application of ultrasound would be effective in lambsquarters. Based on a review of past research, it was found that no information is available evaluating the ability of ultrasound technology to break seed dormancy of common lambsquarters. Therefore, the purpose of this study was to use ultrasound waves as a nondestructive, simple, and low‐cost test to break dormancy in common lambsquarters.

## 2 MATERIALS AND METHODS

### 2.1 Collecting seeds

In this research, common lambsquarters seeds were collected from Moghan Agricultural and Natural Resources Research Field, Iran.

### 2.2 Pretreatment of seeds

Samples were pretreated with produced waves using ultrasound bath (a Bandelin DT 255 H model with internal dimensions of 325 × 175 × 305 mm and volume of 5.5 L). This device is capable of producing ultrasonic waves at the frequency of 35 kHz and the power of 230 W. The ultrasound bath tank was first filled with two liters of distilled water. Then, four replicates of samples were exposed to ultrasound waves with at five time lengths of 0 (control sample), 5, 10, 15, and 30 min.

### 2.3 Measuring germination indicators

After each ultrasound treatment, 50 common lambsquarters seeds were sterilized and placed in a Petri Dish. Then, 10 ml of distilled water was added to the samples and they were put in a germinator at the temperature of 20°C (ISTA, 1996). Germinated seeds were counted every 24 hr for 2 weeks, and germination percentage of the samples was measured. Plumule and radicle were measured using a digital caliper. After 2 weeks (14 days) dry weight of common lambsquarters seedlings was measured by removing 10 samples from each petri dish to an oven at 70°C for 24 hr. Moisture and dry matter in the common lambsquarters seeds were measured using Equations 1 and 2 (Jahanbakhshi, Abbaspour‐Gilandeh, Ghamari, & Heidarbeigi, 2019; Jahanbakhshi, Abbaspour‐Gilandeh, & Gundoshmian, 2018; Jahanbakhshi, Rasooli Sharabiani, Heidarbeigi, Kaveh, & Taghinezhad, 2019; Jahanbakhshi, Yeganeh, & Shahgoli, 2019).

(1) $$\text{MC}=\frac{M_{w}-M_{d}}{M_{w}}\times 100$$

(2) $$\text{DM}=\frac{M_{d}}{M_{w}}\times 100$$

where MC is the moisture content of seed (%), *M*_w_ is the initial mass of seed (mg), *M*_d_ is the mass of dried seed (mg), and DM is the dry matter seed (%).

The Seedling vigor index I (SVI) and II (SVII) of common lambsquarters seed were calculated through Equations 3 and 4 (Bajji, Kinet, & Lutts, 2002).

(3) SVI=GP×SL

(4) SVII=GP×SDW

where SL is the seedling length, SDW is the seedling dry weight and GP is the germination percentage.

### 2.4 Statistical analysis

In this experiment with a randomized complete design was used to analyze the effect of ultrasound at five levels (0 or control, 5, 10, 15, and 30 min) with four replications (20 treatments). The effect of ultrasound on parameters (GP, SL, SDW, SVI, and SVII) was analyzed. SAS 9.4 software was used for analysis and to perform statistical operations. Means of the treatments were compared based on LSD test (*p* ≤ .05; Jahanbakhshi & Kheiralipour, 2019).

## 3 RESULTS AND DISCUSSION

**FIGURE 1:**
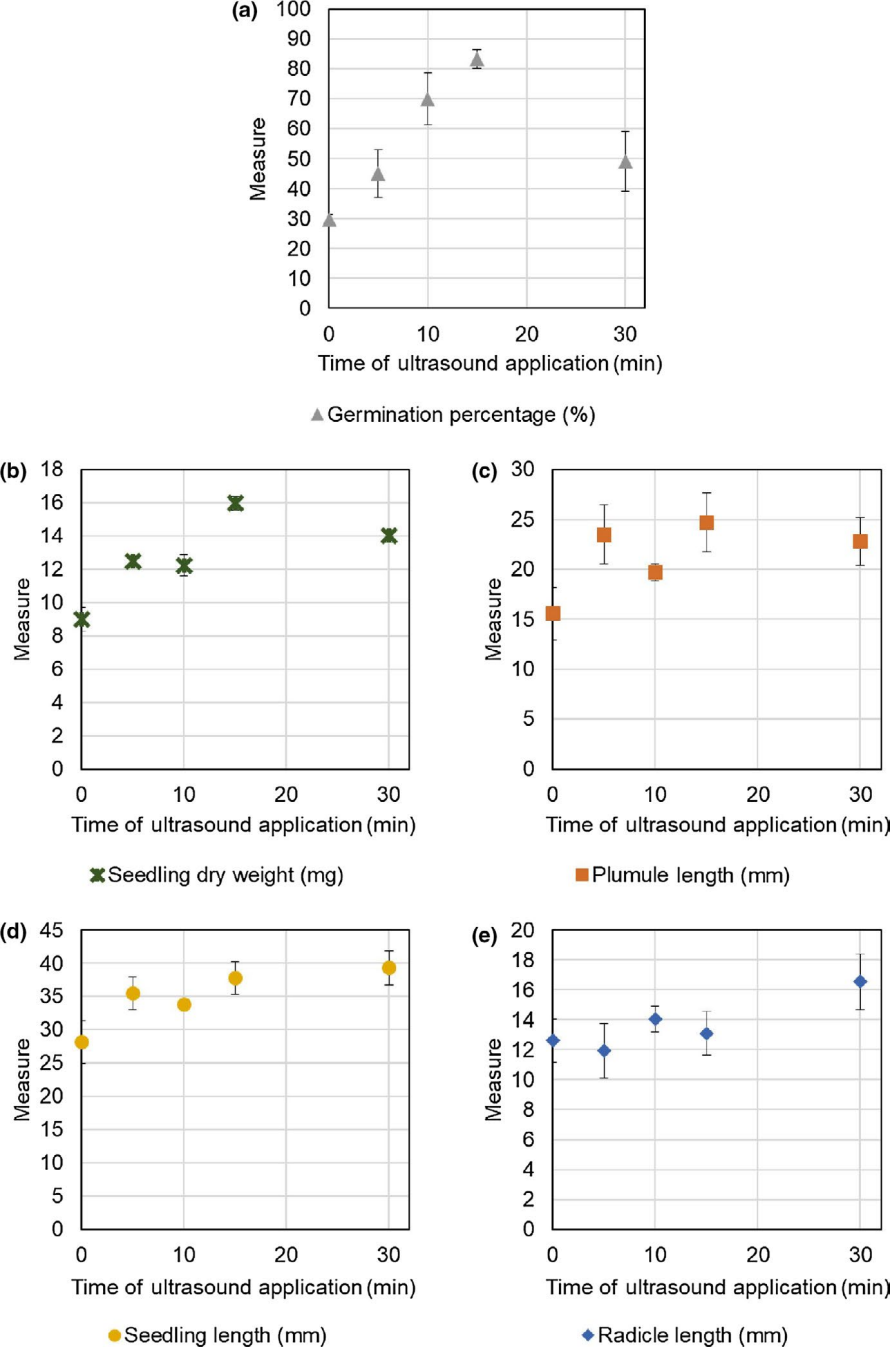
Effect of pretreatment with ultrasound on common lambsquarters (*Chenopodium album*) germination percentage and other commonly measured seedling traits. Data are averages ± standard error of four treatments. Each treatment was composed of 50 common lambsquarters seeds measured 2 weeks after ultrasound treatment applied for the time indicated. Germination indices are (a) Germination Percentage, (b) Seedling Dry Weight (mg), (c) Plumule Length (mm), (d) Seedling Length (mm), and (e) Radicle Length (mm)

The results of analysis of variance for the effect of ultrasound waves on weed germination indices of common lambsquarters are reported in Table 1, and the data are presented in Figure 1 and further analyzed in Table 2.

**TABLE 1:** Analysis of variance of ultrasound waves effects on the germination indices of common lambsquarters

Source of variations	*df*	Germination percentage (%)	Seedling dry weight (mg)	Plumule length (mm)	Seedling length (mm)	Radicle length (mm)	SVI	SVII
Treatment	4	0.658**	0.187**	0.155^ns^	0.072*	0.069^ns^	1.011**	1.417**
Error	15	0.076	0.008	0.064	0.019	0.050	0.093	0.097
CV	—	7.02	3.57	8.40	3.99	8.68	4.09	4.84

Abbreviation: ns, not‐significant.

* Significant at 5% probability level.

** Significant at 1% probability level.

**TABLE 2:** Comparison of the ultrasound mean effects on the germination indices of common lambsquarters

Ultrasonic pretreatments (min)	Germination percentage (%)	Seedling dry weight (mg)	Plumule length (mm)	Seedling length (mm)	Radicle length (mm)	SVI	SVII
0	29.75 c	9.00 d	15.55 b	28.15 b	12.60 a	843.2 c	270.0 c
5	45.00 c	12.50 bc	23.50 a	35.45 ab	11.95 a	1,598.4 bc	562.0 bc
10	70.00 ab	12.25 c	19.70 ab	33.75 ab	14.05 a	2,352.0 ab	861.0 b
15	83.25 a	16.00 a	24.70 a	37.10 a	13.10 a	3,125.6 a	1,328.8 a
30	49.00 bc	14.00 b	22.80 ab	39.35 a	16.55 a	1,921.8 b	697.0 b

Note

Means followed by the same letters in the same column do not have a significant difference based LSD multiple range test at 5% level.

### 3.1 Germination percentage (GP)

Pretreating common lambsquarters seed with ultrasound increases their germination percentage (GP; Tables 1 and 2, Figure 1a). The percentage of germination in the control sample (without ultrasound, 29.75%) was the lowest. The highest germination percentage was observed in pretreatment with ultrasound for 15 min (83.25%). Pretreatment with 5 min is not statistically different from control. Thirty minutes of ultrasound treatment promoted germination more than the control but to a lesser extent than 10 or 15 min. Because of the decrease in percent germination at 30 min compared with 15 min, there was no linear relationship between increasing the ultrasound application time and percent germination. These results are similar to those reported by Sharififar, Nazari, and Asghari (2015). The germination of barley (*Hordeum vulgare*) and fennel (*Foeniculum vulgare*) can also be improved after ultrasound and increasing the duration of ultrasound application reduced the percentage of germination (Fateh, Noroozi, Farbod, & Gerami, 2012; Yaldagard et al., 2008). Other researchers reported that increasing the ultrasound application time from 10 to 20 min to break dormancy in the wild yam (*Dioscorea* spp.) seed yielded unfavorable results and led to decrease in germination percentage (Andriamparany & Buerkert, 2019).

### 3.2 Seedling dry weight (SWD), plumule length (PL), seedling length (SL), and radicle length (RL)

Once the seed has geminated, it can then extend beyond the confines of the seed coat and transition into a seedling. We therefore measured whether pretreatment with ultrasound would have effects on other commonly measured early seedling traits. We found that pretreating common lambsquarters seed ultrasound alters the seedling dry weight, and the length of the plumule and seedling (Tables 1 and 2, Figure 1).

Seedling dry weight common lambsquarters showed a significant difference compared with untreated seeds after the ultrasound pretreatments (Tables 1 and 2, Figure 1b). The smallest seedling dry weight belonged to the control sample (9.00 mg). The largest seedling dry weight (16.00 mg) was observed in 15 min of ultrasound treatment where dry weight of the seedlings increased by 36.1% compared with the control sample. Similar to germination percentage and plumule length, increasing the time of ultrasound application (30 min) reduced seedling dry weight compared with 15 min (Table 2, Figure 1). In similar studies conducted on myrtle (*Myrtus communis*), researchers reported that ultrasound application, caused an increase in radicle and plumule dry weight in addition to breaking dormancy (Alvandian, Vahedi, & Taghizadeh, 2013).

Plumule length of common lambsquarters showed a significant difference compared with untreated seeds after the ultrasound pretreatments (Tables 1 and 2, Figure 1d). The longest plumule length was observed in the 15 min of ultrasound treatment (24.70 mm), and the shortest was seen in the control sample (15.50 mm). In 15‐ and 30‐min ultrasound treatments, the seedling length increased 58.8% and 46.6%, respectively, over the control. The pattern for promotion of plumule length is similar to seedling length (Table 2, Figure 1) where the measure at 10 min of ultrasound pretreatment is less than that at 5 or 15 min (Table 2, Figure 1c). Pretreatment with ultrasound lead to a 27%–59% increase in plumule length. Researchers have reported that the use of ultrasound technology breaks down the cell wall in the seeds. Breaking the cell wall in the seeds increases the water uptake in the cell and ultimately increases the germination and its indices (such as plumule length; Baker, Robertson, & Duck, 2001; Patero & Augusto, 2015).

Seedling length of common lambsquarters showed a significant difference compared with untreated seeds when ultrasound pretreatments were used (Tables 1 and 2, Figure 1c). The longest seedling length of common lambsquarters was seen after 30 min of ultrasound treatment (39.35 mm), and the shortest length was for the control sample (28.18 mm). The trend shows a roughly linear increase in length with increasing time with a 20%–40% increase over the control samples. In similar studies, researchers reported that using ultrasound increased the radicle of barley for 13%–16% compared with the control sample (Yaldagard et al., 2008).

Pretreating common lambsquarters seed with ultrasound does not result in a statistical difference for radicle length. (Tables 1 and 2, Figure 1e). Although pretreatment with 30 min of ultrasound does result in a 1.4‐fold increase in radicle length, the differences are not statistically different. This is because of the high degree of variability seen between the replicated samples.

### 3.3 Seedling vigor index I (SVI) and II (SVII)

The results of analysis of variance showed that seedling vigor index I (SVI) and II (SVII) of common lambsquarters showed significant differences at 1% probability level (Table 1). The highest vigor longitudinal and vigor weighted indices were obtained at 15 min of ultrasound treatment. By increasing ultrasound application time to 30 min, the SVI and SVII decreased and the lowest amount of them was observed in the control sample (Table 2). In 15 min of ultrasound treatment, the SVI and SVII of common lambsquarters increased by 64% and 80%, respectively, in comparison with the control sample. The results presented here are consistent with the findings of other researchers (Machikowa, Kulrattanarak, & Wonprasaid, 2013; Risca, Fartais, & Stiuca, 2007; Yaldagard et al., 2008).

### 3.4 Potential mechanism for ultrasound effect and usefulness of ultrasound applications

Many plants regulate dormancy by imposing physical dormancy. Physical dormancy occurs when the seed coat is impermeable to water and/or gasses that are required for germination. Ultrasound may be promoting germination in common lambsquarters, because it is breaking the physical dormancy. One of the mechanisms through which ultrasound is said to exert its effects is the change that it creates in the plasma membrane to facilitates the entrance and exit of water and mineral elements into cells (Miano et al., 2015). Increasing the activity of alpha‐amylase enzyme and, as a result, increasing the rate of starch hydrolysis, is another effect of ultrasound waves (Kratovalieva et al., 2012). Ultrasound brings about microscopic cracks in the seeds' skin, which facilitates water absorption, and accelerates the germination process (Qin, Xu, Zhong, & Alfred, 2012).

Another type of dormancy is physiological dormancy. Physiological dormancy prevents germination from occurring unless certain chemical changes occur and is the most common type of dormancy (Finch‐Savage & Leubner‐Metzger, 2006). Physical and physiological dormancy do occur together resulting in combinational dormancy; this happens in *Geranium robertianum* (Vandelook & van Assche, 2010). It is known that *Chenopodium* species exhibit physiological dormancy, and the depth of dormancy depends on the seed coat's thickness, which in turn is determined by the environment experienced by the mother plant during seed maturation (Penfield & MacGregor, 2017 and references therein). Breaking physiological dormancy in nature happens through many routes including elevated or fluctuating temperatures, which could be taken to extreme with fire or freezing/thawing events, drying, or passage through the digestive tracts of animals. Breaking physiological dormancy in the laboratory is often accomplished through scarification, after‐ripening in dry storage, and cold or warm stratification (Finch‐Savage & Leubner‐Metzger, 2006). Therefore, it may be that the ultrasound is sufficiently altering the chemistry of the seeds to promote germination and the other traits measured.

As this method requires soaking seeds in water and immersing them in the ultrasound bath, it is unlikely that it can be used in the field to promote germination from the weed seed bank to control plants in the field. Therefore, instead we propose that this method will be useful for researchers who are trying to understand the ecophysiological characteristics of weed seeds. A better understanding of the behaviors and characteristics of weed seeds will allow for better design of effective weed control programs. Therefore, when researchers are looking for differences between treatments, or wild populations, it is essential to be able to experimentally determine the maximum value that can be obtained, particularly with regards to germination percentage as all of the other traits measured herein, or on any seedling, depend on whether or not the seed has germinated. Pretreatment with ultrasound promotes germination, as well as seedling dry weight and the length of the plumule and seedling; if researchers want to study the germination indicators other than germination percentage, further work will be required to determine if the effects are separable.

Although common lambsquarters is a problematic weed, it also has some medicinal uses and is eaten as a leafy vegetable (Choudhary, 2020) and has other potential applications in rangeland/pasture rehabilitation, soil conservation, and animal husbandry. Therefore, when studying lambsquarters for its good as well as its bad properties, it will be important to promote synchronous germination to a high percentage. Ultrasound may play a role in accomplishing this goal.

## 4 CONCLUSION

In this study, we investigated whether ultrasound pretreatment could induce dormancy break in common lambsquarters. Germination percentage, seedling length, seedling dry weight, and seedling vigor index I and II were measured. Different lengths of the ultrasound pretreatment were provided to determine when the maximum dormancy break could be obtained. The results showed that ultrasound pretreatment of common lambsquarters can increase most of the germination indices measured. The highest and lowest germination percentages were equal to 83.25% and 29.75%, respectively, which belonged to the 15 min of ultrasound treatment and the control sample, respectively. As a consequence of the increased germination or independently of it, other seedling measures were also altered in response to the ultrasound treatment. Maximum seedling length (39.35 mm) was observed after 30 min of ultrasound treatment which showed a 40% increase compared with the control sample. Seedling dry weight and the seedling vigor index I and II of common lambsquarters were promoted most by the 15‐min ultrasound treatment, and there was a significant difference between the treated samples and the control sample for all other timepoints. The results of this study showed that by using ultrasound technology as a non‐destructive method, the percentage of germination can be improved in common lambsquarters seeds. Ultrasound is therefore an effective way to increase germination percentage in common lambsquarters and to induce the maximum increase, a pretreatment time of 15 min is recommended.

## CONFLICT OF INTEREST

The authors have declared no conflict of interest.

## ETHICAL APPROVAL

This study does not involve any human or animal testing.

## INFORMED CONSENT

Written informed consent was obtained from all study participants.

## References

[fsn31547-bib-0001] Abbaspour‐Gilandeh, Y. , Kaveh, M. , & Jahanbakhshi, A. (2019). The effect of microwave and convective dryer with ultrasound pre‐treatment on drying and quality properties of walnut kernel. Journal of Food Processing and Preservation, 43(11), e14178 10.1111/jfpp.14178

[fsn31547-bib-0002] Alboresi, A. , Gestin, C. , Leydecker, M. T. , Bedu, M. , Meyer, C. , & Truong, H. N. (2005). Nitrate, a signal relieving seed dormancy in Arabidopsis. Plant, Cell and Environment, 28(4), 500–512. 10.1111/j.1365-3040.2005.01292.x 16229082

[fsn31547-bib-0003] Alebrahim, M. T. , Rashedmihasel, M. H. , Meighani, F. , & Baghestani, M. A. (2011). Study of dormancy‐breaking and optimum temperature for germination of Russian knapweed (*Acroptilon repens* L.). Journal of Plant Protection, 24(4), 391–397.

[fsn31547-bib-0004] Alshallash, K. S. (2018). Germination of weed species (*Avena fatua*, *Bromus catharticus*, *Chenopodium album* and *Phalaris minor*) with implications for their dispersal and control. Annals of Agricultural Sciences, 63(1), 91–97.

[fsn31547-bib-0005] Alvandian, S. , Vahedi, A. , & Taghizadeh, R. (2013). The study on the effect of ultrasonic waves on seed germination in *Myrtus communis* . Seed Research (Journal of Seed Science and Technology), 3(3), 21–31.

[fsn31547-bib-0006] Andriamparany, J. N. , & Buerkert, A. (2019). Effect of ultrasonic dormancy breaking on seed germination and seedling growth of three wild yam species (*Dioscorea* spp.) from SW‐Madagascar. Genetic Resources and Crop Evolution, 66(6), 1167–1174. 10.1007/s10722-019-00779-5

[fsn31547-bib-0007] Asfaram, A. , Sadeghi, H. , Goudarzi, A. , Kokhdan, E. P. , & Salehpour, Z. (2019). Ultrasound combined with manganese‐oxide nanoparticles loaded on activated carbon for extraction and pre‐concentration of thymol and carvacrol in methanolic extracts of *Thymus daenensis*, *Salvia officinalis*, *Stachys pilifera*, *Satureja khuzistanica*, and *mentha*, and water samples. Analyst, 144(6), 1923–1934. 30688952 10.1039/c8an02338g

[fsn31547-bib-0008] Bajji, M. , Kinet, J. M. , & Lutts, S. (2002). The use of the electrolyte leakage method for assessing cell membrane stability as a water stress tolerance test in durum wheat. Plant Growth Regulation, 36(1), 61–70.

[fsn31547-bib-0009] Baker, K. G. , Robertson, V. J. , & Duck, F. A. (2001). A review of therapeutic ultrasound: Biophysical effects. Physical Therapy, 81(7), 1351–1358. 11444998

[fsn31547-bib-0010] Choudhary, V. (2020). Medicinal uses of *Chenopodium album* (Lambsquarters) Bathua. Available from: http://natureconservation.in/medicinal‐uses‐of‐chenopodium‐album‐lambsquarters‐bathua/. Accessed March 6, 2020.

[fsn31547-bib-0011] Chu, T. M. , Jusaitis, M. , Aspinall, D. , & Paleg, L. G. (1978). Accumulation of free proline at low temperatures. Physiologia Plantarum, 43(3), 254–260.

[fsn31547-bib-0012] Cumming, B. G. (1963). The dependence of germination on photo‐period, light quality, and temperature in *Chenopodium* spp. Canadian Journal of Botany, 41, 1211–1233.

[fsn31547-bib-0013] Ding, J. , Hou, G. G. , Dong, M. , Xiong, S. , Zhao, S. , & Feng, H. (2018). Physicochemical properties of germinated dehulled rice flour and energy requirement in germination as affected by ultrasound treatment. Ultrasonics Sonochemistry, 41, 484–491. 29137779 10.1016/j.ultsonch.2017.10.010

[fsn31547-bib-0014] Fallahi, N. , Babaei Ghaghelestany, A. , Asadi Gakieh, M. , & Hatami Gharah Ghovini, N. (2016). The effect of halo‐priming on germination indices of wheat under salinity stress. Agroecology Journal, 11(4), 25–34.

[fsn31547-bib-0015] Fateh, E. , Noroozi, H. , Farbod, M. , & Gerami, F. (2012). Assessment of Fennel (*Foeniculum vulgare*) seed germination characteristics as influenced by ultrasonic waves and magnetic water. European Journal of Experimental Biology, 2(3), 662–666.

[fsn31547-bib-0016] Finch‐Savage, W. E. , & Leubner‐Metzger, G. (2006). Seed dormancy and the control of germination. New Phytologist, 171(3), 501–523. 16866955 10.1111/j.1469-8137.2006.01787.x

[fsn31547-bib-0018] Goussous, S. J. , Samarah, N. H. , Alqudah, A. M. , & Othman, M. O. (2010). Enhancing seed germination of four crop species using an ultrasonic technique. Experimental Agriculture, 46(2), 231–242.

[fsn31547-bib-0019] Graeber, K. , Nakabayashi, K. , Miatton, E. , Leubner‐Metzger, G. , & Soppe, W. (2012). Molecular mechanisms of seed dormancy. Plant, Cell and Environment, 35, 1769–1786. 10.1111/j.1365-3040.2012.02542.x 22620982

[fsn31547-bib-0021] Henson, I. E. (1970). The effects of light, potassium nitrate and temperature on the germination of *Chenopodium album* L. Weed Research, 10, 27–39.

[fsn31547-bib-0022] Herron, J. W. (1953). Study of seed production, seed identification and seed germination of *Chenopodium* spp. Memoirs of Cornell University Agricultural Experiment Station, 320, 1–24.

[fsn31547-bib-0023] Holm, R. E. , & Miller, M. R. (1972a). Weed seed germination responses to chemical and physical treatments. Weed Science, 20, 150–152.

[fsn31547-bib-0024] Holm, R. E. , & Miller, M. R. (1972b). Hormonal control of weed seed germination. Weed Science, 20(3), 209–212.

[fsn31547-bib-0026] Humphries, T. , Chauhan, B. S. , & Florentine, S. K. (2018). Environmental factors effecting the germination and seedling emergence of two populations of an aggressive agricultural weed; *Nassella trichotoma* . PLoS ONE, 13(7), e0199491. 29975730 10.1371/journal.pone.0199491 PMC6033418

[fsn31547-bib-0027] ISTA (1996). International rules for seed testing. Seed Science and Technology, 13, 299–513.

[fsn31547-bib-0028] Jahanbakhshi, A. , Abbaspour‐Gilandeh, Y. , Ghamari, B. , & Heidarbeigi, K. (2019). Assessment of physical, mechanical, and hydrodynamic properties in reducing postharvest losses of cantaloupe (*Cucumis melo* var. *Cantaloupensis*). Journal of Food Process Engineering, 42(5), e13091 10.1111/jfpe.13091

[fsn31547-bib-0029] Jahanbakhshi, A. , Abbaspour‐Gilandeh, Y. , & Gundoshmian, T. M. (2018). Determination of physical and mechanical properties of carrot in order to reduce waste during harvesting and post‐harvesting. Food Science and Nutrition, 6(7), 1898–1903. 10.1002/fsn3.760 30349679 PMC6189625

[fsn31547-bib-0030] Jahanbakhshi, A. , & Kheiralipour, K. (2019). Influence of vermicompost and sheep manure on mechanical properties of tomato fruit. Food Science and Nutrition, 7(4), 1172–1178. 10.1002/fsn3.877 31024690 PMC6475754

[fsn31547-bib-0031] Jahanbakhshi, A. , Rasooli Sharabiani, V. , Heidarbeigi, K. , Kaveh, M. , & Taghinezhad, E. (2019). Evaluation of engineering properties for waste control of tomato during harvesting and postharvesting. Food Science and Nutrition, 7(4), 1473–1481. 10.1002/fsn3.986 31024721 PMC6475739

[fsn31547-bib-0032] Jahanbakhshi, A. , Yeganeh, R. , & Shahgoli, G. (2019). Determination of mechanical properties of banana fruit under quasi‐static loading in pressure, bending, and shearing tests. International Journal of Fruit Science, 1–9. (In press). 10.1080/15538362.2019.1633723

[fsn31547-bib-0033] Karssen, C. M. , Brinkhorst‐van der Swan, D. L. C. , Breekland, A. E. , & Koornneef, M. (1983). Induction of dormancy during seed development by endogenous abscisic acid: Studies on abscisic acid deficient genotypes of *Arabidopsis thaliana* (L.). Heynh. Planta, 157(2), 158–165. 10.1007/bf00393650 24264070

[fsn31547-bib-0034] Kaveh, M. , Jahanbakhshi, A. , Abbaspour‐Gilandeh, Y. , Taghinezhad, E. , & Moghimi, M. B. F. (2018). The effect of ultrasound pre‐treatment on quality, drying, and thermodynamic attributes of almond kernel under convective dryer using ANNs and ANFIS network. Journal of Food Process Engineering, 41(7), e12868 10.1111/jfpe.12868

[fsn31547-bib-0035] Kępczyński, J. , & Sznigir, P. (2013). Response of *Amaranthus retroflexus* L. seeds to gibberellic acid, ethylene and abscisic acid depending on duration of stratification and burial. Plant Growth Regulation, 70(1), 15–26.

[fsn31547-bib-0036] Kratovalieva, S. , Srbinoska, M. , Popsimonova, G. , Selamovska, A. , Meglič, V. , & Anđelkovic, V. (2012). Ultrasound influence on coleoptile length at Poaceae seedlings as valuable criteria in prebreeding and breeding processes. Genetika, 44(3), 561–570.

[fsn31547-bib-0037] Liu, J. , Wang, Q. , Karagić, Đ. , Liu, X. V. , Cui, J. , Gui, J. , … Gao, W. (2016). Effects of ultrasonication on increased germination and improved seedling growth of aged grass seeds of tall fescue and Russian wildrye. Scientific Reports, 6, 22403. 26928881 10.1038/srep22403 PMC4772161

[fsn31547-bib-0038] Long, Y. , Tan, D. Y. , Baskin, C. C. , & Baskin, J. M. (2012). Seed dormancy and germination characteristics of *Astragalus arpilobus* (Fabaceae, subfamily Papilionoideae), a central Asian desert annual ephemeral. South African Journal of Botany, 83, 68–77.

[fsn31547-bib-0039] Machikowa, T. , Kulrattanarak, T. , & Wonprasaid, S. (2013). Effects of ultrasonic treatment on germination of synthetic sunflower seeds In Proceedings of World Academy of Science, Engineering and Technology (No. 73, p. 53). USA: World Academy of Science, Engineering and Technology (WASET).

[fsn31547-bib-0040] Majd, R. , Aghaie, P. , Monfared, E. K. , & Alebrahim, M. T. (2013). Evaluating of some treatments on breaking seed dormancy in Mesquite. International Journal of Agronomy and Plant Production, 4(7), 1433–1439.

[fsn31547-bib-0041] Miano, A. C. , Forti, V. A. , Abud, H. F. , Gomes‐Junior, F. G. , Cicero, S. M. , & Augusto, P. E. D. (2015). Effect of ultrasound technology on barley seed germination and vigour. Seed Science and Technology, 43(2), 297–302.

[fsn31547-bib-0042] Moechnig, M. J. , Stoltenberg, D. E. , Boerboom, C. M. , & Binning, L. K. (2003). Empirical corn yield loss estimation from common lambsquarters (*Chenopodium album*) and giant foxtail (*Setaria faberi*) in mixed communities. Weed Science, 51(3), 386–393.

[fsn31547-bib-0043] Patero, T. , & Augusto, P. E. (2015). Ultrasound (US) enhances the hydration of sorghum (*Sorghum bicolor*) grains. Ultrasonics Sonochemistry, 23, 11–15. 25465094 10.1016/j.ultsonch.2014.10.021

[fsn31547-bib-0044] Penfield, S. , & King, J. (2009). Towards a systems biology approach to understanding seed dormancy and germination. Proceedings of the Royal Society B: Biological Sciences, 276(1673), 3561–3569. 10.1098/rspb.2009.0592 PMC2817297 19605392

[fsn31547-bib-0045] Penfield, S. , & MacGregor, D. (2017). Effects of environmental variation during seed production on seed dormancy and germination. Journal of Experimental Botany, 68(4), 819–825. 10.1093/jxb/erw436 27940467

[fsn31547-bib-0046] Qin, P. , Xu, L. , Zhong, W. , & Alfred, C. H. (2012). Ultrasound‐microbubble mediated cavitation of plant cells: Effects on morphology and viability. Ultrasound in Medicine and Biology, 38(6), 1085–1096. 22502880 10.1016/j.ultrasmedbio.2012.02.017

[fsn31547-bib-0047] Rifna, E. J. , Ramanan, K. R. , & Mahendran, R. (2019). Emerging technology applications for improving seed germination. Trends in Food Science and Technology, 86, 95–108.

[fsn31547-bib-0048] Risca, I. M. , Fartais, L. , & Stiuca, P. (2007). Ultrasound effects contributions on the Norway spruce seeds germination (*Picea abies* (L.) Karsten). Genetics and Molecular Biology, 8, 87–88.

[fsn31547-bib-0049] Sadeghianfar, P. , Nazari, M. , & Backes, G. (2019). Exposure to ultraviolet (UV‐C) radiation increases germination rate of maize (*Zea maize* L.) and sugar beet (*Beta vulgaris*) seeds. Plants, 8(2), 49. 10.3390/plants8020049 PMC6409551 30813484

[fsn31547-bib-0050] Schuster, C. L. , Shoup, D. E. , & Al‐Khatib, K. (2007). Response of common lambsquarters (*Chenopodium album*) to glyphosate as affected by growth stage. Weed Science, 55(2), 147–151.

[fsn31547-bib-0051] Sharififar, A. , Nazari, M. , & Asghari, H. R. (2015). Effect of ultrasonic waves on seed germination of *Atriplex lentiformis*, *Cuminum cyminum*, and *Zygophyllum eurypterum* . Journal of Applied Research on Medicinal and Aromatic Plants, 2(3), 102–104.

[fsn31547-bib-0052] Shekari, F. , Mustafavi, S. H. , & Abbasi, A. (2015). Sonication of seeds increase germination performance of sesame under low temperature stress. Acta Agriculturae Slovenica, 105(2), 203–212.

[fsn31547-bib-0053] Tang, D. S. , Hamayun, M. , Ko, Y. M. , Zhang, Y. P. , Kang, S. M. , & Lee, I. J. (2008). Role of red light, temperature, stratification and nitrogen in breaking seed dormancy of *Chenopodium album* L. Journal of Crop Science and Biotechnology, 11(3), 199–204.

[fsn31547-bib-0054] Vandelook, F. , & Van Assche, J. A. (2010). A combined physical and physiological dormancy controls seasonal seedling emergence of *Geranium robertianum* . Plant Biology, 12, 765–771. 10.1111/j.1438-8677.2009.00290.x 20701699

[fsn31547-bib-0055] Williams, J. T. (1962). Dormancy in *Chenopodium album* L. The Annals of Applied Biology, 50, 352.

[fsn31547-bib-0056] Williams, J. T. (1963). Biological flora of the British Isles, *Chenopodium album* L. The Journal of Ecology, 51, 711–725.

[fsn31547-bib-0057] Yaldagard, M. , Mortazavi, S. A. , & Tabatabaie, F. (2008). Influence of ultrasonic stimulation on the germination of barley seed and its alpha‐amylase activity. African Journal of Biotechnology, 7(14), 2465–2471.

